# The dilemma of improving rational antibiotic use in pediatric community-acquired pneumonia

**DOI:** 10.3389/fped.2023.1095166

**Published:** 2023-02-08

**Authors:** Phuong T. K. Nguyen, Paul D. Robinson, Dominic A. Fitzgerald, Ben J. Marais

**Affiliations:** ^1^ Department of General Medicine, The Children’s Hospital Westmead, Westmead, NSW, Australia; ^2^ Department of Respiratory Medicine, The Children’s Hospital Westmead, NSW, Australia; ^3^ The University of Sydney, Discipline of Child and Adolescent Health, Faculty of Medicine and Health, Sydney, NSW, Australia; ^4^ Department of Infectious Diseases, The Children’s Hospital Westmead, Westmead, NSW, Australia

**Keywords:** acute respiratory infections, lower respiratory tract infections, pneumonia, antibiotic, antimicrobial resistance

## Abstract

Pneumonia is the number one cause of disease and deaths in children under five years old, outside the neonatal period, with the greatest number of cases reported from resource-limited settings. The etiology is variable, with not much information on the local etiology drug resistance profile in many countries. Recent studies suggest an increasing contribution from respiratory viruses, also in children with severe pneumonia, with an increased relative contribution in settings that have good vaccine coverage against common bacterial pathogens. Respiratory virus circulation was greatly reduced during highly restrictive measures to contain the spread of COVID-19 but rebounded once COVID-19 restrictions were relaxed. We conducted a comprehensive literature review of the disease burden, pathogens, case management and current available prevention of community acquired childhood pneumonia, with a focus on rational antibiotic use, since the treatment of respiratory infections is the leading cause of antibiotic use in children. Consistent application of revised World Health Organisation (WHO) guidance that children presenting with coryzal symptoms or wheeze can be managed without antibiotics in the absence of fever, will help to reduce unnecessary antibiotic use, as will increased availability and use of bedside inflammatory marker tests, such as C–reactive protein (CRP) in children with respiratory symptoms and fever.

## 1. Introduction

Deaths among children under 5 years old mostly occur in the neonatal period (36.2%), but pneumonia (12.6%) and diarrhea (9.7%) remain the main cause of mortality in children under-5 years old in the post-neonatal period (1). Childhood pneumonia epidemiology, etiology and case management are variable in different WHO regions (2), with most deaths occurring in resource-limited settings where there is a limitation on antibiotics access (which is the mainstay treatment for bacterial pneumonia), on wide uptake of routine vaccination, on improving hygiene and nutrition, as well as on the availability of oxygen therapy (3). However, inappropriate antibiotic use is a global concern with alarming rates of antimicrobial resistance (AMR) documented in many regions, especially in resource-limited settings with limited microbiology services and extensive empiric antibiotic use (4). Lower respiratory tract infections is a major driver of inappropriate antibiotic use (4, 5).

Recent studies demonstrated that viruses contribute more to lower respiratory tract infections than previously appreciated, also in developing country settings and in children with severe disease (6–8). Strict social restrictions implemented to contain the COVID-19 pandemic and use of personal protective equipment (PPE) reduced respiratory virus transmission and associated secondary bacterial pneumonia, as demonstrated by a significant decrease in influenza infections and pneumonia due to *Streptococcus pneumoniae* in children (9, 10). However, major increases in respiratory virus infections were documented in many settings once COVID-19 social restrictions were relaxed. Australia experienced unprecedented and unseasonal respiratory syncytial virus (RSV) outbreaks during 2021 and 2022 (11).

The use of antibiotics is often promoted to reduce death from respiratory infections, but unnecessary and poorly targeted antibiotic use may do more harm than good (12, 13). Therefore, carefully considering the risks and benefits of antibiotic use in any individual patient is important. While appropriately used antibiotics will reduce morbidity and mortality from bacterial pneumonia, inappropriate use contributes to rising rates of AMR. With increased awareness, AMR is now recognized as a major global threat that has an impact on incidence, death, hospital stay and health care costs in a wide range of human diseases, including child pneumonia (4, 14). In many instances antibiotics are used inappropriately due to health system factors or parental expectations (4, 5, 15). There is a great need for doctors and health care systems to promote responsible antibiotic use by implementing effective antibiotic stewardship approaches (5). We conducted a comprehensive literature review of pneumonia epidemiology, aetiology and management with a focus on rational antibiotic use in childhood pneumonia.

## 2. Global pneumonia disease burden

**Figure 1:**
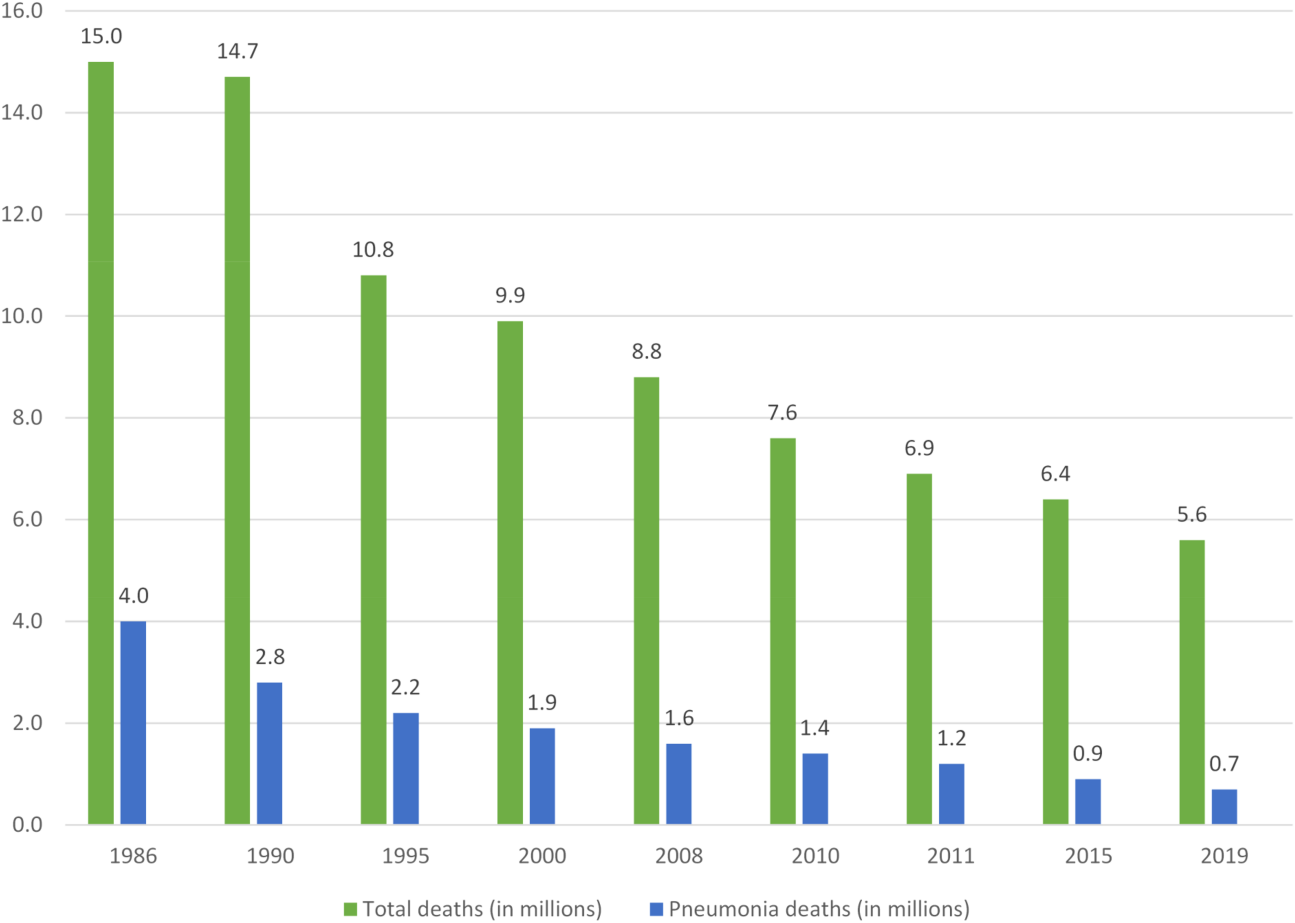
Declining total and pneumonia-related deaths in children under five years of age*. Percentages represent pneumonia deaths as a proportion of total deaths. *Adapted from (3, 14, 91)

In 2019, pneumonia was responsible for an estimated 0.7 (0.6–0.8) million deaths in children under five years old (16), with the highest number of pneumonia episodes per child year reported in North Africa and the Middle East. Although deaths related to child pneumonia has reduced over last 10 years, mortality remains high in many areas. Children in sub-Saharan Africa experience the highest mortality, accounting for 44.6% (312,400 deaths) of all pneumonia-related deaths in children under five (17). Table 1 illustrates pneumonia morbidity and mortality in different World Health Organisation (WHO) regions in 2016—the latest year for which regional data are available. Globally, both developing and developed country settings have achieved substantial reductions in pneumonia disease burden since the turn of the century (1, 18). Over this period the contribution of pneumonia to all-cause mortality declined from 16.6% [uncertainty range (UR): 14.8–17.9%] to 14.0% (UR: 12.0–15.1%) with the largest reduction in South Asia, from 17.5% (UR: 15.2–20.6%) to 11.7% (UR: 9.6–14.4%), mainly due to the implementation of expanded vaccination programs (16). Figure 1 summaries the decreasing trend of global child pneumonia mortality among children under five years old from 1986 to 2019.

**Table 1:** Estimated regional and global pneumonia disease burden in children under five.

Region	Estimated pneumonia disease burden*			
	Deaths	Death per 100,000 children	Millions of episodes	Episodes per 1,000 children
Central and eastern Europe, central Asia	17,000	60.4	3.0	107.1
Latin America and Caribbean	21,800	44.0	4.7	94.9
Southeast Asia, east Asia and Oceania	60,200	48.9	14.8	120.4
North Africa and middle east	39,700	62.9	8.4	133.2
South Asia	199,500	129.9	18.8	122.1
Sub-Saharan Africa	312,400	199.5	15.8	100.6
Global	652,600	103.3	68.1	107.7

* Estimates for 2016 (17).

The declines in child pneumonia morbidity and mortality were related to improved socioeconomic conditions and the application of routine vaccinations in the world, resulting in better coverage with measles, *Haemophilus influenzae* type b (Hib) and pneumococcal vaccines (PCV), as well as better access to healthcare systems and the effective case management application (16, 19). Hib vaccine is now delivered in all countries around the world as part of the WHO Expanded Programme of Immunisation (EPI). Its positive impact is well recognized and Hib deaths declined significantly (78%–96%) after more than 10 years of vaccine coverage in countries where these vaccines were included in routine vaccination schedules (19). As of the end of 2021, PCV had been introduced in most countries, except Somali, Guinea, South Sudan, Chad, Cabo Verde, Comoros, Equatorial Guinea, Gabon and Egypt (20). In 2022, Somalia, Guinea, South Sudan and Chad introduced PCV for the first time (20). Interestingly, PCV immunization studies in South Africa demonstrated an associated significant reduction (31%, 95% CI: 15%–43%) in the incidence of pneumonia hospitalization associated with common respiratory viruses (influenza A/B, parainfluenza types 1–3, RSV, adenovirus) (21). The mechanism remains unclear, but this could be the result of indirect protection *via* trained innate immunity induction. Currently, PCV-13 and PCV-10 are available for prevention of childhood pneumonia. PCV-7 was first licensed but was rapidly replaced by the higher-valent PCV-10 that covered additional invasive pneumococcal serotypes. Currently, PCV-13 is used most widely in routine immunisation programs, offering protection against all PCV-10 serotypes as well as serotypes 3, 6A, and 19A (22).

## 3. Common causes of community-acquired pneumonia

Childhood pneumonia is caused by many pathogens, including viruses, bacteria, mycobacteria (mainly *Mycobacterium tuberculosis*), fungi, and parasites (8). It is not easy to confirm the cause of a child pneumonia case (5) due to difficulties in obtaining the respiratory samples, especially in small children, the low sensitivity and uncertain specificity of microbiological investigation (23). In addition, a detected pathogen is sometimes hard to conclude as a true cause of disease due to the risk of contamination with upper airway flora, especially with non-invasive specimen collection methods (24). Disease causing pathogens are influenced by the child's age, geographic locality, and vaccination coverage. Recent studies from multiple settings have identified respiratory viruses as the most common cause of community acquired pneumonia in young children (6, 8). Table 2 provides an overview of common pathogens associated with paediatric pneumonia in otherwise healthy children. In neonates and children with immune compromise, congenital abnormalities or bronchiectasis, additional pathogens require consideration.

**Table 2:** Common pathogens associated with community acquired pneumonia in children*.

Categories	Pathogens
Viruses	Respiratory syncytial virus (RSV)
	Rhinovirus (serotypes A, B, C)
	Influenza virus (A, B)
	Parainfluenza virus (serotypes 1–4)
	Human metapneumovirus (A, B)
	Adenovirus (multiple serotypes)
Bacteria	*Streptococcus pneumoniae*
	*Staphylococcus aureus*
	*Haemophilus influenzae* type b^a^
	Nontypable *H. influenzae*
Mycobacteria	*Mycobacterium tuberculosis*
Atypical bacteria	*Mycoplasma pneumonia* ^b^
	*Chlamydia pneumoniae* ^b^

^a^ In settings with poor vaccination coverage.

^b^ Mostly in children older than 5 years of age.

* Outside of the neonatal period and excluding risk groups (adapted from (25).

### 3.1. Viruses

With increased uptake of routine Hib and pneumococcal vaccines, respiratory viruses have become the main pathogens associated with pneumonia; both severe and non-severe (8). The most commonly identified viruses in all age groups include RSV, rhinovirus, as well as influenza and parainfluenza viruses (26, 27).

#### 3.1.1. Respiratory syncytial virus (RSV)

The most common virus causing pediatric pneumonia in the multi-country Pneumonia Etiology Research for Child Health **(**PERCH) study was RSV, accounting for 31.1% of all cases; more than three times as common as any other pathogen (8). The clinical manifestations of RSV infection range from mild upper respiratory infections to severe pneumonia with respiratory failure. Common sequelae include prolonged wheezing with hyper-reactive airways (28). RSV is commonly associated with co-infection with other respiratory viruses and bacteria, which complicates aetiological determination (7, 8). Social distancing measures to contain COVID-19 disrupted RSV transmission with greatly reduced respiratory disease burden, but once social distancing was relaxed it returned with even greater prevalence and virulence. It has been postulated that this reflects reduced population immunity after a period of limited transmission, referred to as “immune debt” (11, 29). RSV is a major vaccine development target and promising new candidate vaccines will enter phase 3 trials in the near future (30).

#### 3.1.2. Rhinoviruses and influenza viruses

Rhinoviruses have been detected with increasing frequency in children diagnosed with pneumonia (31) and are traditionally associated with common cold symptoms and frequently detected in asymptomatic individuals (mean prevalence 15%; up to 60%–80% in children). This is significantly more than other common respiratory viruses (prevalence 1%–5%) (32), which complicates interpretation of the role of rhinoviruses as a pneumonia pathogen (33). In immunocompromised hosts severe pneumonia has been attributed to rhinovirus infections (34). The current rhinovirus classification identifies three serotypes (A, B, C), which are further divided into 169 subtypes. A study from three sub-Saharan Africa countries conducted in children 13–59 months of age, reported an association between rhinovirus C and wheezing disease (35), but type specific differences have not been consistently observed.

The contribution of influenza viruses to childhood pneumonia is often under-appreciated. Studies have demonstrated that severe pneumococcal pneumonia is also more common in the presence of influenza infection (Odds Ratio (OR): 3.7; 95% Confidence Interval (CI): 1.0–18.1) and if not given universally pneumococcal vaccine should be considered in children with recurrent influenza virus infections (36). The most effective way to reduce the impact of influenza infection is seasonal vaccination (37), which was first recommended for health care workers to reduce their risk of contracting disease, with less time off work and reduced risk of spreading it to vulnerable patients within health care facilities (38). Influenza vaccine given to pregnant women was shown to provide protection to their newborn babies (49% protection documented in a study from South Africa), a particularly vulnerable group (39), and the United States Centers for Disease Control (CDC) was the first major public health agency to recommend universal seasonal vaccination (40).

#### 3.1.3. Other viruses

Adenovirus-associated pneumonia is relatively uncommon (documented in 2%–12% of pneumonia cases), but it can cause severe necrotising pneumonia (especially serotypes 3, 7, and 14) (41, 42). A post-mortem study conducted in China among 175 children who died with pneumonia found that 9% of pulmonary tissue specimens were positive with adenovirus (43). Human metapneumovirus has been identified in 12% of children admitted with pneumonia in the United States (44); with detection in 44% of children under 12 months admitted with pneumonia in Spain (45). The prevalence of human metapneumovirus was ≥10% among children hospitalised with pneumonia in 4/7 study sites in the PERCH study (8). In a study from Thailand human bocavirus was the third most common pathogen detected, after rhinovirus and RSV, accounting for 12% of all cases (46). SARS-CoV-2, the cause of the COVID-19 pandemic, also infects children, but it rarely causes severe disease (47) and this risk is further reduced by vaccination (48). Four other coronaviruses are established human pathogens, but only cause mild cold like symptoms (49).

### 3.2. Bacterial pathogens

Studies showed that bacterial pathogens were more detected in severe childhood pneumonia which often required hospitalisation (8). However, the role of bacteria in less severe disease remains controversial with more potential causes identified when more tests are done. A recent study that applied a variety of detection tests such as antibody tests for *S. pneumoniae* and *H. influenzae*, as well as sensitive polymerase chain-reaction (PCR) and solid-phase immunoassays for respiratory viruses, identified potential pathogens in 85% of pneumonia cases (50). Co-infection of bacteria with respiratory viruses was seen in 41%, but it is unclear how to interpret these microbial associations and how it should guide antibiotic treatment (51). Historic lung aspirate and autopsy studies have confirmed the importance of *S. pneumoniae, Staphylococcus aureus and H. influenzae*, including non-typable strains, as causes of severe lobar pneumonia (8, 52).

#### 3.2.1. Streptococcus pneumoniae

*S. pneumoniae* remains a leading cause of bacterial pneumonia, meningitis, and sepsis in children worldwide (53), despite the availability of effective vaccines and antibiotic treatment (8). *S. pneumoniae* causes around 11% (8%–12%) of all deaths in children less than 5 years [excluding pneumococcal deaths in human immunodeficiency virus (HIV) positive children], with most (61%) pneumococcal deaths occurring in ten African and Asian countries (53). Although lobar consolidation is typically associated with bacterial pneumonia, lobar consolidation is relatively uncommon among pneumonia cases (54–56) and is observed with both viral and bacterial infection.

#### 3.2.2. Haemophilus influenzae

The contribution of *H. influenzae* decreased significantly after widespread implementation of routine conjugated Hib vaccine (57). Following vaccine roll-out studies in Africa and China, Hib became an uncommon cause for pneumonia and was less common than non-typeable *H. influenzae* (8)—which was reported at an increased frequency in some studies (58, 59). A lung aspirate study among severe pneumonia children from The Gambia found that 23% of samples were positive with *H. influenza*; non-typeable *H. influenza* accounted for 5% of all bacteria isolated (60).

#### 3.2.3. Other bacteria

Atypical bacteria such as *Mycoplasma pneumoniae (M. pneumoniae)* and *Chlamydia pneumoniae (C. pneumoniae)* appear to be important pathogens in school-age children. A multi-center study from Italy assessing routine nasopharyngeal aspirates samples collected in 613 children 2–14 years old admitted with pneumonia found *M. pneumoniae* in 34.3% and *C. pneumoniae* in 14.1% of patients (61). Similar to respiratory viruses, its aetiologic role is confounded by frequent asymptomatic infection (62). Non-typhoidal salmonella has also been documented as an important cause of childhood pneumonia and sepsis, but mainly in malaria endemic areas (63). Children with hospital-acquired pneumonia (which per definition occurs ≥48 h after hospital admission) are often infected with gram-negative bacilli or *S. aureus* (64). *S. aureus* is also seen in community acquired pneumonia, particularly in adolescent children, and can follow an aggressive course if treatment is delayed or sub-optimal, which is common with methicillin-resistant *S. aureus* (MRSA) infection (8). *S. aureus* and *Pseudomonas aeruginosa* are also problematic pathogens in children with bronchiectasis, including cystic fibrosis (CF) (65). Interestingly, RSV infection has been shown to promote *P. aeruginosa* colonisation in this setting (66). In neonates *group B streptococcus*, *Escherichia coli* and *Chlamydia trachomatis* require consideration (67).

#### 3.2.4. Mycobacterium tuberculosis

In high tuberculosis (TB) incidence settings, *M. tuberculosis* has been identified as a cause or co-pathogen in 5%–10% of children with severe pneumonia (8). Very young, malnourished and HIV-infected children are particularly vulnerable to develop pneumonia due to TB (6). Although traditionally associated with more chronic symptoms, TB can present as acute severe pneumonia with clinical and radiographic features indistinguishable from other causes of pneumonia (68). However, children with recent close TB contact or who live in a TB endemic setting and experience persistent symptoms, such as cough, fever, reduced playfulness and/or weight loss that do not respond to initial treatment should be suspected of TB (69, 70).

## 4. Pneumonia management

Due to challenges in determining child pneumonia etiology, differences in environmental risk factors and inconsistent uptake of effective vaccines against common pathogens around the world, pneumonia case management is variable in different settings. The need for a simple case management approach to guide care and improve outcomes in resources-limited settings has been recognised (71). The WHO developed and published a simple case management approach based on clinical signs and symptoms to recognize severe conditions (cases with any danger sign) that require urgent stabilization and guide the management of children with severe and non-severe pneumonia. Hypoxaemia management is important and appropriate oxygen therapy can reduce child pneumonia deaths (72). A study that introduced oximetry and oxygen therapy in five district hospitals in Papua New Guinea, a high-burden setting for child pneumonia, found a 35% reduction in deaths in children hospitalised with severe pneumonia (73).

### 4.1. Appropriate antibiotic use

**Figure 2:**
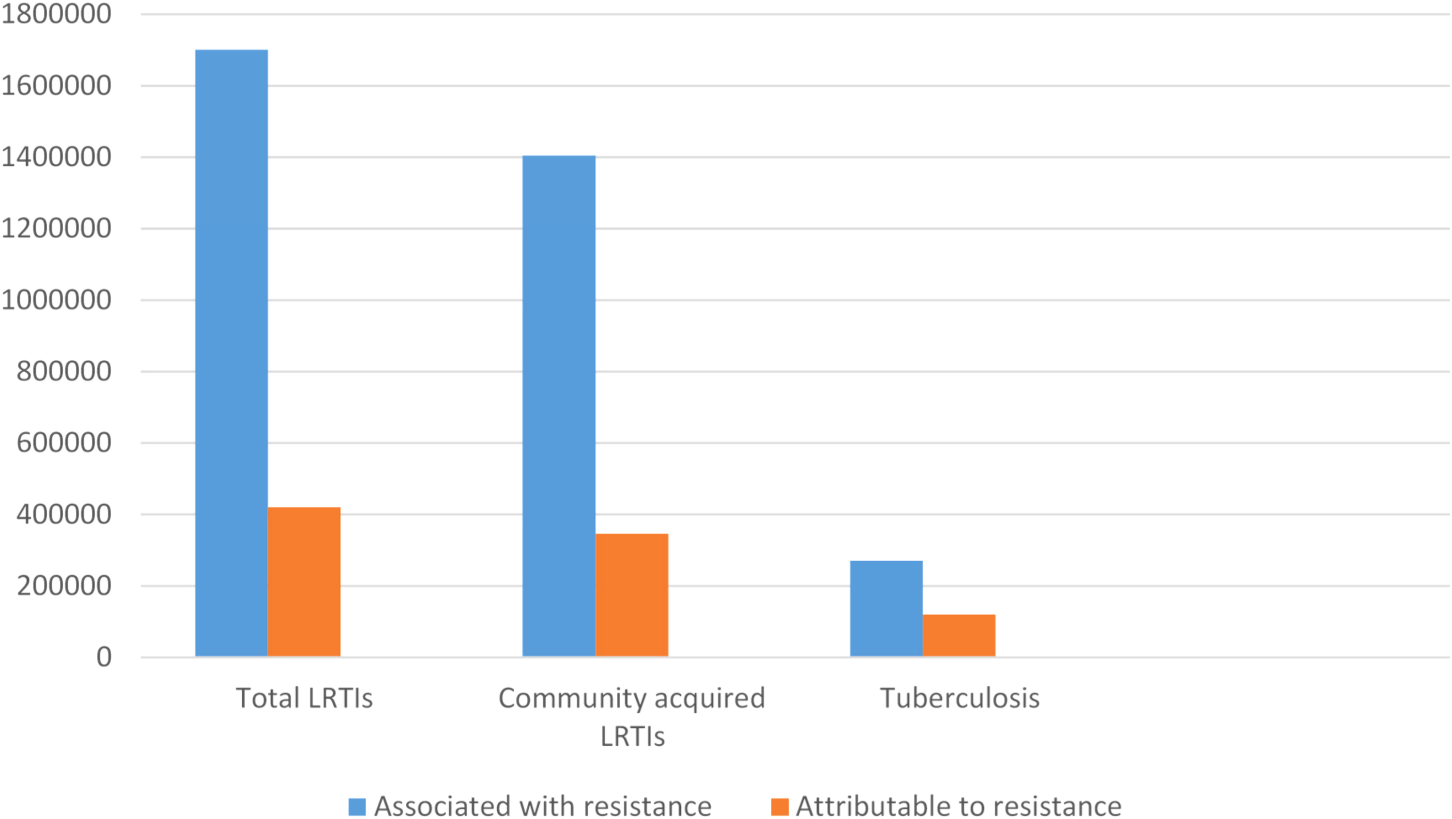
Estimated global deaths counts associated with and attributable to antimicrobial resistance in people with lower respiratory tract infection and tuberculosis in 2019*. *Adapted from (4). LRTIs, lower respiratory tract infection.

Acute respiratory infections is a major reason for antibiotic use in children (74). Excessive and irrational antibiotic use is well documented all over the world and contributes to antimicrobial resistance (AMR) within the community. Figure 2 illustrates the number of deaths attributed to AMR infections globally. Although rising AMR rates pose a major health care threat, its value in reducing pneumonia deaths in children needs to be recognized. Use of standardised case management approaches that included antibiotic use reduced under five pneumonia deaths by 70% in developing country settings (75). However, given excessive hospital admission and use of parenteral antibiotics, WHO revised the clinical criteria for “severe” pneumonia so that those with fast breathing and/or chest in-drawing alone were no longer classified as “severe” pneumonia and could receive oral antibiotics as outpatients (67). This revision greatly reduced the annual incidence of severe pneumonia hospitalisations in infants, ranging from a difference of −301.0/100,000 infants (95% confidence interval, CI: −405.2 to −196.8) in Fiji to −3242.6/100,000 infants (95% CI: −3695.2 to −2789.9) in The Gambia, without a negative impact on patient outcomes (76).

A major dilemma is the need to ensure that all children with bacterial pneumonia receive antibiotics to reduce pneumonia-related deaths, but at the same time to ensure that children with viral infections and conditions such as bronchiolitis or asthma do not receive inappropriate antibiotics. With good PCV and Hib vaccine coverage an increasing proportion of child pneumonia cases is caused by viral infections (8). Excessive use of empirical antibiotics, especially with broad-spectrum agents, has been recognised as a particular problem in Asian countries. Children with mild respiratory symptoms and no clinical evidence of severe pneumonia often receive empiric broad spectrum antibiotics without clinical indication (77). In addition, there is easy and unregulated access to a wide range of antibiotics, unlike developing countries where access is often limited or developed country settings where access is regulated. Table 3 provides a summary of risks and benefits of antibiotic treatment in paediatric pneumonia. It is important for clinicians to be familiar with the most common bacterial causes, their local drug resistance profiles and the likely positive and negative impacts of antibiotic therapy.

**Table 3:** Summary of benefits and risks of antibiotic use in children with pneumonia.

Benefits	Risks
Reduced morbidity & mortality if bacterial pneumonia	Increases antimicrobial resistance (AMR) with cumulative risk for the individual patient and the community
No direct benefit in viral infections, although it may reduce secondary bacterial infections (especially with severe disease) (78)	Disturbs the microbiome with potential long term impacts on microbiome composition, growth and metabolism, especially if used early in life (94)
Often meets parental expectations; reduces clinician & parental anxiety, as well as perceived medicolegal risk	Admission for intravenous antibiotic administration may result in unnecessary hospitalisation with associated cost and risk of line associated complications, as well as nosocomial infection (79)
Use of oral antibiotics can keep children out of hospital	Regular and prolonged empiric use of broad-spectrum antibiotics increases the risk of drug resistance and harm. This is an even greater risk in resource limited settings with poor access to microbiology services.
Cheap and easy to give.	Inappropriate empiric antibiotic use may provide a false sense of security, which may delay accurate diagnosis

A major difficulty in child pneumonia management is knowing when to safely withhold antibiotic treatment in a child with suspected pneumonia based on clinical evaluation (3). In developed countries, the available of point-of-care diagnostic tests to distinguish between viral and bacterial infections and identify the causative pathogen(s) include a full blood count, C-reactive protein (CRP), and a viral panel test have been found to be useful in guiding antibiotic treatment. However, microbiological confirmation of the causative agent remains challenging, with high rates of co-infection complicating the picture (24). The PERCH study found that elevated CRP ≥40 mg/l was positively associated with confirmed bacterial pneumonia (especially *S. pneumoniae* *and* *H. influenzae*) and negatively associated with RSV (80). A recent study in Vietnam demonstrated that a point-of-care CRP test, using a cut-off of <50 mg/l, safely reduced antibiotic use in children with an acute respiratory infection presenting to primary healthcare centres (68.5% antibiotic use within 2 weeks follow up vs. 76.7% in control group, *p* = 0.0001) (81). Wheeze has been associated with viral infections in both African (82) and Asian settings (83). Revised WHO guidance recommends that a child with wheeze and no fever, in the absence of any WHO danger sign, should not receive antibiotic treatment (84). In those with fever or without a wheeze, a CXR and full blood count (and/or CRP) could provide clinicians with additional confidence to withhold antibiotics in a child with acute respiratory symptoms (85). Table 4 summarises some difficult dilemmas where the relevant trade-offs with antibiotic use is difficult.

**Table 4:** Scenarios presenting difficult dilemmas in antibiotic use.

Scenarios	Considerations
Pro-active antibiotic use in vulnerable children with immune compromise where a viral infection may trigger a serious bacterial superinfection, or in children admitted with severe viral pneumonia in whom bacterial superinfection cannot be ruled out.	Empiric antibiotics is warranted in any serious clinical condition, but should be stopped as soon as a bacterial infection/superinfection can be ruled out. Excessive antibiotic exposure will only select resistant bacteria. With severe viral infection it is important to be vigilant of bacterial superinfection, but more evidence is required to define appropriate empiric antibiotic use in this group (12, 13).
Parental demand for antibiotics when not considered appropriate eg. in a child with a runny nose and wheezing	Important to educate parents and the general public about the harms associated with antibiotic use, including AMR risk; particularly in communities where antibiotic use has been normalized (5, 86).
Uncomplicated community acquired pneumonia potentially caused by bacteria, where empiric antibiotic use has demonstrated benefit.	Treat with oral amoxicillin for a duration of 3-5 days (67). However, the optimal duration of first-line therapy remains debated and is the topic of ongoing study.

AMR, antimicrobial resistance; MRSA, methicillin-resistant Staphylococcus aureus; HIV, human immunodeficiency virus.

## 5. Pneumonia prevention

Childhood and maternal immunisations against *S. pneumoniae*, *Hib*, *Bordetella pertussis*, measles and influenza viruses are highly effective prevention strategies, similar to the encouragement of exclusive breastfeeding during the first 6 months of life; ideally maintained for 1–2 years. Other important prevention strategies include reducing cigarette smoke exposure in young children, with reduced indoor and outdoor air pollution where this is a concern. Improving maternal health education, reductions in unnecessary hospitalisations and enhanced infection control in health care facilities to reduce the risk of nosocomial infections are additional prevention methods to reduce childhood pneumonia globally (87, 88).

Prophylactic antibiotic use can also be considered in specific high-risk cases. *Pneumocystis jirovecii* pneumonia (PJP) is preventable by cotrimoxazole preventive therapy and in HIV-infected children effective anti-retroviral therapy is most important (89). Like any antibiotic, cotrimoxazole prophylaxis also increases drug resistance (90, 91). Study in Botswana found that children using cotrimoxazole prophylaxis developed amoxicillin resistance (91). Given amoxicillin is first-line medication for management of “non-severe” pneumonia cases in primary settings around the work, this finding is concerning. Moreover, amoxicillin has been assigned to an “access category” by the WHO AWaRe antibiotic classification (67, 92). Cotrimoxazole prophylaxis also increase resistance to other antibiotics, such as chloramphenicol and ciprofloxacin (93).

### Broader clinical considerations

This review describes the dilemma of appropriate antibiotic use in childhood community acquired pneumonia, given the challenge of rising antibiotic resistance rates around the world. A detailed discussion of complimentary treatment and the management of immunocompromised children is not included. As a narrative review it is open to subjectivity and bias, but every effort was made to provide a balanced overview and to include all the most relevant references. Data on the global pneumonia disease burden dates from 2016 is presented, which is somewhat dated, but it represents the latest published data and should provide an accurate overview of the situation before 2019. No reliable global data has been compiled since the COVID-19 pandemic. Our discussion of childhood community acquired pneumonia management only includes antibiotic and oxygen use, since these are the areas with the best available evidence and antibiotic treatment is the focus of our review. The intent was to provide an up-to-date, concise and informative overview stimulating critical assessment of appropriate antibiotic use, as encouraged by the new WHO AWaRe initiative (86).

## 6. Conclusion

Pneumonia remains the leading cause of mortality and morbidity in childhood, but this can be greatly reduced with good prevention and case management strategies. With increasing vaccine coverage respiratory viruses are now recognised as major pathogens, which requires a reconsideration of optimal antibiotic use. Appropriate antibiotic treatment saves lives, but it is important to limit inappropriate antibiotic use that drives the emergence of antimicrobial resistance without clinical benefit. Guidance about when and which antibiotics to use will be highly dependent on the local context, but it is important for clinicians to carefully consider the relevant trade-offs that should inform their decision.
